# The European antibody network's practical guide to finding and validating suitable antibodies for research

**DOI:** 10.1080/19420862.2015.1100787

**Published:** 2015-09-29

**Authors:** Giovanna Roncador, Pablo Engel, Lorena Maestre, Amanda P. Anderson, Jacqueline L. Cordell, Mark S. Cragg, Vladka Č. Šerbec, Margaret Jones, Vanda J. Lisnic, Leonor Kremer, Demin Li, Friedrich Koch-Nolte, Núria Pascual, Jose-Ignacio Rodríguez-Barbosa, Ruurd Torensma, Helen Turley, Karen Pulford, Alison H. Banham

**Affiliations:** 1 Monoclonal Antibody Unit; Spanish National Cancer Research Center; Madrid, Spain; 2 Immunology Unit, Department of Cell Biology; Immunology and Neurosciences; Medical School, University of Barcelona; Spain; 3 NDCLS, Radcliffe Department of Medicine; University of Oxford; Oxford, UK; 4 OxFabs; University of Oxford; Oxford UK; 5 Antibody and Vaccine Group; Cancer Sciences Unit; University of Southampton; Faculty of Medicine; General Hospital, Southampton, UK; 6 Center for the Production of Diagnostic Reagents and for Research; Blood Transfusion Center of Slovenia; Ljubljana, Slovenia; 7 Center for Proteomics; Faculty of Medicine; University of Rijeka; Rijeka, Croatia; 8 Department of Immunology and Oncology; Spanish National Center for Biotechnology; Consejo Superior de Investigaciones Científicas (CNB/CSIC); Madrid, Spain; 9 Institute of Immunology; University Medical Center; Hamburg, 20246 Germany; 10 Custom Antibody Service (CAbS); IQAC-CSIC/CIBER-BBN; Barcelona, Spain; 11 Section of Immunobiology; Institute of Biomedicine; University of Leon; Léon, Spain; 12 Department of Tumorimmunology; Radboud University Medical Center; Nijmegen, The Netherlands

**Keywords:** BLAST searches, CiteAb, EuroMAbNet, flow cytometry, gene silencing, HLDA workshops, immunohistochemistry, monoclonal antibodies, validation studies, western blotting

## Abstract

Antibodies are widely exploited as research/diagnostic tools and therapeutics. Despite providing exciting research opportunities, the multitude of available antibodies also offers a bewildering array of choice. Importantly, not all companies comply with the highest standards, and thus many reagents fail basic validation tests. The responsibility for antibodies being fit for purpose rests, surprisingly, with their user. This paper condenses the extensive experience of the European Monoclonal Antibody Network to help researchers identify antibodies specific for their target antigen. A stepwise strategy is provided for prioritising antibodies and making informed decisions regarding further essential validation requirements. Web-based antibody validation guides provide practical approaches for testing antibody activity and specificity. We aim to enable researchers with little or no prior experience of antibody characterization to understand how to determine the suitability of their antibody for its intended purpose, enabling both time and cost effective generation of high quality antibody-based data fit for publication.

## Abbreviations

BLAST: Basic Local Alignment Search Tool
CD: Cluster of Differentiation
c-FLIP: cellular FLICE-like inhibitory protein
ELISA: enzyme-linked immunoabsorbent assay
FCM: flow cytometry
FOXP1: forkhead box protein P1
HLDA: Human Leukocyte Differentiation Antigens Workshop
IP: immunoprecipitation
NCBI: National Center for Biotechnology Information
SDS-PAGE: sodium dodecyl sulfate polyacrylamide gel electrophoresis.

## Introduction

The ability of antibodies to bind specifically and with high affinity to their target antigens has led to the widespread exploitation of these reagents within the scientific, clinical and commercial communities. Antibodies are commonly used as research tools to study the expression, localization or function of their targets, and they are one of the most prolific classes of new biologics for clinical therapy, particularly in the fields of oncology, rheumatology, transplantation and hematology.^1^ Given the importance of these reagents and their impact within the scientific and medical communities, it is absolutely imperative that all researchers are confident that any antibodies they use are of the correct specificity. The aim of this review is to share the collective experience of a panel of international experts in antibody production and characterization to enable the research community not only to obtain relevant antibodies but to ensure the validity of these reagents.

Historically, monoclonal antibody production and their characterization was performed mainly by specialized laboratories. Antibodies were generally raised against mixtures of proteins and the exact antigen was often unknown. In 1982, a numbering system of unique clusters, each designated a cluster of differentiation (CD), for identifying antibody reactivity against epitopes on the surface molecules of leucocytes was devised at the first meeting of the Human Leukocyte Differentiation Antigens (HLDA) Workshop. This system has served successfully as a global antibody classification scheme, and the numbers of CD have risen dramatically during the subsequent 9 HLDA workshops. It was the exchange of antibodies among laboratories and their independent evaluation by several expert research groups using a range of different techniques that enabled the utility and reproducibility of these early reagents to be so well characterized.^2^

During the last decade, the sequencing of the human genome and the success of antibody-based therapeutics have had a profound effect on the production and availability of both polyclonal and monoclonal antibodies. Many academic groups, including large-scale efforts by the Swedish Human Protein Atlas^3,4^ and the German Antibody Factory,^5^ along with commercial companies have adopted a ‘gene-centric’ approach in an effort to generate antibodies to specific target antigens. This has expanded to include antibodies to distinguish isoforms of the same protein, detect post-translationally modified forms, such as protein phosphorylation and, more importantly, those with the ability to modulate biological functions. There has also been a corresponding growth in commercial providers offering customised monoclonal and polyclonal antibody production services. It should be noted that antibodies with functional activity against their target antigen, and those against non-protein targets or post-translational modifications are not considered within the scope of this article. However, many of the principles described below apply equally to these reagents.

High throughput antibody production, and the lucrative returns generated by sales, have led to a flood of antibodies becoming widely available to the research community. There are more than 300 antibody suppliers providing >500,000 antibodies for the research and clinical markets (www.the-scientist.com/?articles.view/articleNo/32042/title/Antibodies-User-Survey/).

Additionally, many antibodies described in the scientific literature, and sold commercially, were produced by academic research groups. While some companies do make substantial efforts to validate their antibodies and provide detailed product literature showing the results, not all antibodies from commercial sources are fully validated and fit for purpose. Researchers are frustrated by the difficulties caused by poorly validated antibodies and sometimes also by batch-to-batch variability. Even the same monoclonal antibody from different suppliers may exhibit variability in performance. This has recently led a coalition of 112 researchers to propose that polyclonal antibodies should be phased out and all monoclonal antibodies should be defined by their antibody gene sequences and expressed as recombinant proteins to enable standardization of reagents.^6^ While this will indeed identify individual antibodies (something that is often impossible between current suppliers), it will not address whether the antibody has the desired activity or specificity. Furthermore, very similar antibodies may be encoded by different sequences (e.g., humanization variants), while different post-translational modifications such as patterns of glycosylation, which can be regulated by the production methodology, can significantly affect the functionality of a therapeutic antibody.

Others in the scientific community have stressed the need to improve antibody validation because of the adverse effect that poor quality reagents have on research budgets, the research time wasted on ineffective experiments and the publication of inaccurate data in the scientific literature.^7-10^ Several academic reviews have discussed how to validate antibodies; however, these have been for specific fields of research, such as neuroscience;^11^ for validation of antibodies to a single protein, e.g., c-FLIP^12^ or to families of proteins, including central nervous system ion channel proteins^13^ and G-protein-coupled receptors;^14^ or validation for specific techniques such as immunohistochemistry,^15-18^ Western blotting (WB),^19^ reverse phase protein arrays^20^ or chromatin immunoprecipitation (ChIP).^21^ There have also been helpful recommendations on how to report research antibody use to increase experimental reproducibility,^22^ and some journals are now adopting antibody validation requirements for their published articles.^23^

In reality, it is not practical to expect antibodies to be validated for all purposes, and even a partially validated antibody to a new antigen is of potential value to the research community. However, there needs be more clarity as to exactly which reagent is being described, its supplier(s) and the validation that has been performed. Unaltered original clone names should be consistently used for monoclonal antibodies and catalog and batch numbers reliably adopted for polyclonal antibodies. Furthermore, the validation data (including images) needs to be available for independent review, either in the published literature or online, including antibody databases and commercial product datasheets. Also, mechanisms must be established that: 1) enable meaningful dialog between antibody suppliers and users; and 2) allow issues about reagents that might be a cause for concern to be raised in a transparent fashion. For example, some companies already encourage and provide online end user reviews of their reagents. Some issues may be reagent based while others may be specific to individual users. Some companies are willing to independently test off-target activities that are identified for their antibodies. In a recent case raised by a EuroMAbNet member, the supplier not only revised the product sheet but also contacted all the clients who had previously purchased the reagent. The emergence of independent third-party test sites that offer antibody validation services indicates that there is a demand for further validation.

EuroMAbNet is the first European network of academic laboratories specialized in the production and use of monoclonal antibodies. The motivation behind the writing of this article is to share our practical experience of antibody production, selection and validation with other members of the research community. This spans several decades, varying experimental techniques and includes the use of both undefined antigens (e.g., cell lysates) and gene-centric approaches to generate antibodies. We have worked with polyclonal and monoclonal antibodies from our own laboratories and from other sources, including academic groups and companies. Furthermore, EuroMAbNet members have collaborated with commercial antibody suppliers to license their own antibodies for sale to the research community. This makes us particularly well positioned to appreciate the viewpoints of both the academic community and commercial antibody suppliers.

### Step-by-step guide to antibody selection and validation

**Figure 1.**
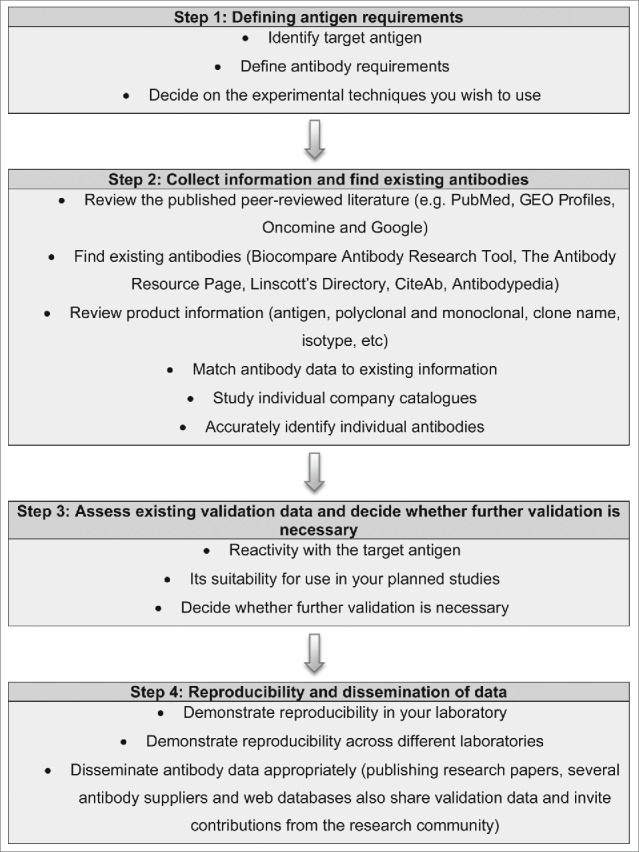
Overview of the fundamental principles involved underlying effective antigen design, antibody selection and validation. A step-by-step guide to defining the target antigen, identifying relevant existing antibodies and their specificity to the accurate dissemination of data arising from their use.

Here, we provide our overview to the fundamental principles underlying effective antigen design, antibody selection and validation (**Fig. 1**). This information is provided in a format to help researchers who need to source or validate antibodies across a broad spectrum of research fields and technical applications. However, it is important to emphasize that there are many widely used well-characterized antibodies that require no additional validation. This is generally evident from their frequent citation in the scientific literature and their well-populated product information sheets. CiteAb is particularly helpful for identifying these reagents, which are ranked by their frequency of literature citation (www.citeab.com/). However, it should be noted that citations accrue over time, thus the initial antibodies generated against an antigen may have accumulated citations (sometimes these reagents may even be suboptimal but were used due to the lack of alternatives) while there may be newer and more effective reagents available that inevitably have fewer citations. However, researchers should always err on the side of caution and confirm that prior validation has been performed, particularly if intending to use these antibodies in new techniques, tissues or different species. Web-based validation guidelines providing additional video tutorials and illustrated examples, including an antibody catalog with further validation files, are available at www.euromabnet.com.

### STEP 1: Define your initial requirements

When sourcing antibodies, decisions must first be made about the: 1) target antigen, 2) necessary antibody characteristics, and 3) proposed technical applications (for a summary and useful websites, see **Table 1**). Many antibodies are purchased purely because the manufacturer recommends them for a particular application, while antibodies that might be more specific but were not extensively tested are ignored. Some basic bioinformatics at the outset can save substantial amounts of time and money by enabling prioritization of existing reagents, and the identification of those with the minimal requirement for additional downstream validation.

**Table 1.** Bioinformatics analysis to define target antigen

Identify the target antigen	Be aware that molecules can occasionally share a common name so search for the approved nomenclature at http://www.genenames.org/
Find alternative gene names	Useful for a review of the historical literature https://www.ncbi.nlm.nih.gov/IEB/Research/Acembly/ or http://www.genecards.org/
Obtain ‘canonical’ protein sequence	http://www.uniprot.org/ Information also provided on additional isoforms
Further define antigen restrictions	*E.g.* specificity for, or commonality across: isoforms, functional domains, processed forms, domains with different subcellular localization (*e.g.*, extracellular versus intracellular portion), post-translational modifications
Is cross species recognition of the antigen critical?	Use BLAST to identify protein sequence identity across orthologous species http://blast.ncbi.nlm.nih.gov/Blast.cgi
Search for related proteins	A BLAST search will also identify related proteins that share regions of identity with the target antigen at http://blast.ncbi.nlm.nih.gov/Blast.cgi
Define ideal epitope(s)	Unique regions in the target antigen conferring specificity or sometimes those regions conferring cross reactivity

### 1A. Define the target antigen requirements

Start by identifying the target antigen, its approved nomenclature and any alternative names that might also help identify existing reagents in the scientific literature. Then, obtain the reference (canonical) protein sequence and identify if variants exist, e.g., those produced by alternative splicing, proteolytic cleavage, post-translational modification. Information on known variants is available on the web, e.g., in Aceview, UniProt and the scientific literature. Decide whether it is important to detect all variants or only one in particular, and whether distinguishing domains with different subcellular localizations may also be critical, e.g., labeling the extracellular versus intracellular portion of membrane proteins. For example, the Abnova FOXP1 monoclonal antibody (M01), clone 4E3-G11, (Catalog number H00027086-M01) product sheet described the immunogen as a full-length FOXP1 recombinant protein of 115 amino acids, yet the FOXP1 reference sequence was 677 amino acids. Further validation defined the epitope within the unique C-terminus of FOXP1 isoform 2 and demonstrated this reagent was unable to detect the full-length FOXP1 protein, which exhibited a significantly different expression pattern.^24^

Perform a search of the non-redundant protein sequences using the National Center for Biotechnology Information (NCBI) Basic Local Alignment Search Tool (BLAST; http://blast.ncbi.nlm.nih.gov/Blast.cgi) using the reference protein sequence. Our guidelines include an online tutorial for those with no experience of BLAST (http://www.euromabnet.com/guidelines/example1.php). This simple analysis will enable further identification of protein variants, regions of the protein that are highly conserved in orthologous species and regions that share sequence identity with closely related proteins. This information is key to using this bioinformatics data to help prioritise antibodies for further study. The aim of this process is to define whether there are: 1) distinct regions of the chosen protein containing linear epitopes that are unique to the individual antigen; and 2) regions of the target antigen where antibodies are highly likely to have cross-reactivity with other proteins with which they share sequence identity. Such reagents may be very valuable, but are likely to require more validation of their specificity.

### 1B. Define antibody requirements

Next, determine the necessary antibody requirements, e.g., monoclonal vs. polyclonal antibody, isotype requirements (further information in **Table 2**). While reported activity in a specific technique is often one of the main criteria used to identify antibodies, the intended application may be less important than specificity, particularly when there are no well-characterized antibodies to the target protein. Also, consider whether what is known about the technical applications can help to prioritise reagents for other techniques. For example, an antibody that recognizes a formalin-resistant epitope for immunohistochemistry may also work in another technique using formalin fixation, such as ChIP.

**Table 2.** Determine antibody requirements

Monoclonal (mAb) vs. polyclonal antibody (pAb)	mAb - single epitope, single isotype, unlimited supply of identical reagent, identifiable (usually) by clone name. pAb - multiple epitopes, better for some techniques as recognizing a range of different epitopes can increase the number of suitable technical applications and enhance signals by enabling more antibodies to bind the same antigen molecule and by forming large precipitating lattices. Conversely, there is more risk of cross-reactive epitopes if the immunogen shares identity with other proteins because a polyclonal antibody will recognize a range of different epitopes. Batch variability, caused by limited quantity and differences in the immune response during subsequent production in another animal, requires additional validation. Sometimes it can be hard to conclusively identify reagents used in the literature or distinguish them among those offered by multiple suppliers.
Isotype	Different isotypes may be useful for experiments using multiple antibodies *e.g.* enabling detection using isotype specific secondary reagents. In both *in vitro* and *in vivo* studies, ‘isotype-dependent’ or ‘isotype specific’ antibody functionality may be important.
Host species	As above it can help to have different species for multiple labeling. Also the use of same species antibodies and tissues needs additional strategies to avoid secondary antibodies detecting endogenous immunoglobulins, this can be avoided if the antibody is raised in a different species.
Define intended technical applications	Consider the intended use in technical applications such as IHC, IHC-P, ICC, IF, FCM, ChIP, WB, ELISA, IP. Abs that recognize linear epitopes (anti-peptide Abs) tend to work well for WB and IHC-P. Abs recognizing native epitopes tend to work well for IP, ELISA and FCM (Abs raised against native proteins, cDNA immunization, cell-based immunogens). While antibodies against extracellular epitopes are commonly used for FCM and for antibody therapeutics, FCM is also increasingly used for antibodies recognizing both intracellular and intranuclear epitopes.
Identify individual antibodies	Refine the list of antibodies using their unique identifiers to remove duplicates such as the same antibody being available from different suppliers. Use the clone name to identify monoclonal antibodies. For polyclonal antibodies, bear in mind that those with an identical host species, immunogen and/or images in validation data may be the same reagent.

Immunohistochemistry (IHC), Immunohistochemistry on formalin fixed and paraffin embedded tissues (IHC-P), immunocytochemistry (ICC), Immunofluorescence (IF), flow cytometry (FCM), chromatin immunoprecipitation (ChIP), Western blotting (WB), enzyme-linked immunosorbent assay (ELISA), immunoprecipitation (IP).

Antibodies raised against synthetic peptides recognize a linear epitope. Hence, anti-peptide antibodies usually work well in WB analyses, but not necessarily in assays with native proteins, such as flow cytometry (FCM), ELISA and IP. Conversely, antibodies raised by cDNA or cell immunization or by immunizations with native proteins often work well in FCM, ELISA and IP, but not in WB. With antibodies recognizing a native epitope, special techniques, such as native SDS-PAGE, refolding on nitrocellulose membranes, dot blot, may allow binding on immunoblots.

### STEP 2: Identification and availability of existing antibodies

Having defined the fundamental requirements for the antibody reagent, a variety of methods can then be used to find existing antibodies. The published scientific literature is the best starting place for discovering which antibodies have already been used to study a target protein. If little information is available, then try using the antigen name, the term ‘antibody’ and the required technique in general search engines (e.g., Google) that examine whole manuscripts, commercial datasheets, theses, patents and abstracts. Bear in mind the importance of antibody availability. Despite scientific convention and journal publishing conditions, not all research laboratories will agree to supply reagents. Collaborations with biotechnology or pharmaceutical companies may require very restricted terms and a lot of paperwork. Some batches of polyclonal antibodies may be in short supply or no longer in existence.

Examination of online antibody listings from commercial suppliers may reveal a much wider range of reagents compared with thumbing through one or 2 catalogs available in the laboratory. Start by employing web-based resources Biocompare (www.biocompare.com/Antibodies/), the Antibody Resource Page (www.antibodyresource.com) and Linscott's Directory (www.linscottsdirectory.com), and search tools such as CiteAb that rank antibodies by citations (www.citeab.com) and Antibodypedia (www.antibodypedia.com). Using official nomenclature (www.genenames.org) for the antigen, and also other names in common usage, can increase the number of antibodies identified. Having confidence in the reputation of the supplier may also help when choosing antibodies; an Antibodies User Survey conducted in 2012 by The Scientist and Frost and Sullivan provides some helpful information on the suppliers most frequently used and those with the highest customer ratings (www.the-scientist.com/?articles.view/articleNo/32042/title/Antibodies-User-Survey/).

Start by identifying individual antibodies, bearing in mind that there may be multiple suppliers providing the same reagent (tips on how to do this are detailed in **Table 2**). Each antibody should have a unique identifier that distinguishes it from other antibodies, and this should not be altered or abbreviated by suppliers or users. Abbreviations and changes of clone names make it difficult to link commercial products with published validation of the antibody. Critically, efforts should be made by researchers, peer reviewers and journals to ensure that unique identifiers are used in any publications arising from use of antibody reagents. Currently, many laboratories fail to identify the exact antibody used in their studies and just describe whether it was a monoclonal or polyclonal antibody and the supplier. The supplier may at the time, or in the future, license multiple antibodies to the same antigen making it difficult to source reagents or even reproduce published data.

**Table 3.** Prioritisation of available antibodies

Validation	The ideal situation is to identify a well-characterized antibody that is widely described in the scientific literature and that already fulfils all the necessary technical requirements. Often a more pragmatic approach is needed, balancing likely specificity versus suitability for defined techniques.
Review product data sheets	Assess the quantity and quality of the validation data and whether this has been performed using the preferred technical applications. STEP 4 and **Table 4** provide more information on how to assess antibody validation.
Immunogen	The immunogen is one of the most critical factors determining the specificity and thus likely suitability of an antibody.
Technical applications	Established functionality in the desired technical application is desirable. If an otherwise desirable antibody is not recommended for a technical application it is worth contacting the supplier to find out whether it is known to be unsuitable or whether the absence of data just means it hasn't yet been tested in the application.
Match antibody data with existing information	Even when there is no published expression data in the literature there is likely to be information that is publically available as to where the transcript is expressed. BioGPS provides transcript expression, including meta-analysis of publically available microarray datasets: http://biogps.org/ RNA Seq data from cancer tissues and cell lines is available through cbioportal: http://www.cbioportal.org/public-portal/ Recent drafts of the human proteome provide searchable online resources where protein expression across a range of tissues, determined by mass spectrometry, can be searched. http://www.humanproteomemap.org; http://www.proteomicsdb.org The human protein atlas contains antibody labeling data for many proteins: http://www.proteinatlas.org/index.php This information can be compared to any data presented in a product data sheet.

While monoclonal antibodies are usually referenced by their clone name, many polyclonal antibodies are not adequately identified, and it is possible to unknowingly buy the same reagent from different distributors. There is also rarely any nomenclature used to differentiate between batches of the polyclonal antibody produced in different animals. As the same immunogen can generate very different immune responses in another member of the same animal species, when sourcing polyclonal antibodies it is advisable to find up-to-date references to the reagent and possibly to buy several aliquots from the same batch to ensure consistency during a project.

### STEP 3: Prioritization of candidate antibodies

When there is a well-characterized reagent, or very few available antibodies, then little prioritization is needed prior to obtaining the reagent(s) and starting experimental testing. However, few academic laboratories have the resources to test an extensive panel of antibodies. Be careful to check whether literature references provided on antibody product sheets are actually those using the particular antibody described. Choosing antibodies that are most likely to meet the specific requirements defined in STEP 1 is critical within an extensive panel of reagents, particularly if nothing more than the information provided on the supplier's datasheets is available to guide selection. The following section will describe how to proceed when there is no existing published literature.

Review the product literature to identify the reagents that best match the antibody requirements devised in STEP 1. Pay particular attention to the quantity and quality of the existing antibody validation data (further information on how to approach this is described in STEP 4) and whether it has been performed in the desired species, tissue or application. Does this data fit with what is known about the expression pattern and potential variants of the antigen?

Examining the immunogen can, in many instances, determine which antibodies are likely to have the closest match to the specificity requirements identified from the bioinformatics analyses in STEP 1. Some suppliers do not disclose the immunogen and consider this to be proprietary information. Many researchers feel that suppliers should be required to disclose detailed information on the immunogen used for antibody production, and it has been proposed that withholding this information prevents independent scientific replication of the reagent. There have even been suggestions that these reagents “are not fit for scientific work” and that “work done with them will not be publishable.”^8^ However, in reality, there is no guarantee that the same immunogen will generate a comparable antibody when used to immunise a different animal, and immunization with a mixture of proteins was used to produce many of the widely used CD antibodies.

The key disadvantage of not having specific information regarding the immunogen is that more extensive validation is often necessary to confirm the specificity of antigen recognition. While suppliers may refuse to disclose the exact details of their immunogen, some have been willing to provide additional general information on request. For example, when needing an antibody to target a defined region or to avoid a specific region (e.g., to avoid antigens with high sequence homology), antibody manufacturers are often willing to disclose whether their immunogen maps to these defined regions. It is in their interests for the research community to successfully use their reagents and convince others to do the same by publishing good-quality data.

Bear in mind that antibodies distinguishing protein isoforms might quite legitimately have very different tissue labeling patterns, multiple or variable banding patterns in WB or different subcellular staining. Several published antibody validation schemes suggest discarding reagents that recognize more than a single protein species by WB. Having this information on the protein under study may help to predict the forms of the protein that are likely to be detected by individual antibodies.

Choosing the region of the protein with most sequence conservation (highest identity) increases the chances of antibodies recognizing the target antigen across different species. However, species cross-reactivity must be validated experimentally, particularly if there are differences in the protein sequences. The regulatory T-cell marker FOXP3 is highly conserved across species, and yet one or 2 amino acid differences within epitopes were sufficient to determine whether antibodies are cross-reactive or species specific.^25^

At this point, it is also worth investigating additional antibody validation that may be available through online antibody databases, which are described at the end of STEP 5.

### STEP 4: Antibody Validation

Before using an antibody, without any further independent characterization, it remains ultimately the responsibility of the researcher to review the existing validation data for its specificity for the target antigen. It is essential that this data relates to the methodology being used (as an individual antibody may not be effective across all techniques), the species/tissue type under study and the technical application being used. Detailed practical guides on how to validate antibodies for different technical applications and across species are provided online (www.euromabnet.com/guidelines/), and an overview is described below and in **Table 4**. An isotype and host species matched control antibody, without any reactivity in your experimental system, should also be obtained and used as a negative experimental control for non-specific reactivity.

**Table 4.** Antibody Validation

Reactivity with the immunogen	Usually the initial testing performed during high throughput antibody screening e.g., using ELISA, WB, FCM
Reactivity with the target antigen	Particularly when linear epitopes are used (e.g., peptides) the antibody reactivity needs to be tested against the target protein. Often in an epitope tagged recombinant form.
Reactivity with the endogenous target antigen	Reactivity with a recombinant antigen does not guarantee reactivity with the endogenous protein.
Specificity for the target antigen	Some potential cross reactivity can be predicted based on sequence identity and, if likely, should be experimentally determined. Whenever possible use more than one antibody to define novel expression patterns. Further investigation is required if different reagents give discordant data, with each other or with other sources of information such as transcript expression, or data from other laboratories.
Evidence for suitability in intended application(s)	Reactivity and specificity for the target antigen in one technique or tissue does not guarantee that this will be the same in others. Thus it is important to validate antibodies for additional experimental techniques and the range of tissue types being studied.
Validation for use in orthologous species	The reactivity and specificity for the target antigen should be validated for each specific species being studied.

### STEP 4A. Validation of target antigen recognition

High throughput screening for reactivity with the immunogen using techniques such as ELISA, FCM or WB is usually the starting point for antibody validation. This may be the only form of validation, often in only a single technical application, provided for many antibodies. Custom antibody services commonly guarantee the production of antibodies that recognize the immunogen (particularly by ELISA), as this is achievable for nearly all projects. However, many antibodies that recognize the immunogen then fail to recognize the recombinant or endogenous protein in subsequent testing. This is particularly common if the immunogen: 1) is a short linear epitope (e.g., a synthetic peptide), 2) does not adopt the correct 3-dimensional native conformation, or 3) is produced in a prokaryotic expression system that lacks post-translational modifications (e.g., glycosylation). It is also important to note that the expression level of the endogenous protein is often much lower than that of its recombinant counterpart in transfected cells (the latter commonly being driven by a strong promoter). Moreover, the endogenous protein may be engaged in interactions with other proteins, nucleic acids, or membranes that may partially or fully mask the binding site of an antibody.

Many groups transfect mammalian cell lines that lack or have low levels of the transcript for the target antigen with epitope tagged expression constructs to further test antibody reactivity with the target antigen. VERIFY Antigen Standards™ from Origene now provide more than 10,000 tagged overexpression lysates for antibody validation by WB. This approach can be very helpful as WB enables molecular weight determination and comparison with that of any endogenous proteins that are detected (allowing for any size difference caused by the tag). Immunolabelling techniques such as immunohistochemistry also enable a comparison of the staining by the antibody under study with that from an antibody to the epitope tag to confirm a similar frequency of positive cells and comparable subcellular colocalization of the target antigen. Detection of a tagged protein or co-expression with fluorescent proteins (such as green fluorescent protein) can be used to identify, sort or purify cells expressing the target antigen by FCM, enabling verification that the same population is detected by the antibody being characterized. Multicolor FCM with a panel of markers can also determine whether the antigen is expressed on a rare subpopulation of cells. Labeling cells with and without permeabilization for FCM can also distinguish between antibodies binding antigens with an intracellular versus cell surface localization. Conjugation reactions, for generating directly labeled antibodies, need to be carefully evaluated as they can compromise or change antibody-antigen binding.^26^

Importantly, recognition of a recombinant protein expressed in the appropriate species does not guarantee that the antibody will recognize the endogenous antigen; rare instances where an antibody can recognize the endogenous protein, but not the recombinant antigen, have occurred. Antibodies that fail to recognize the endogenous protein may still be useful research tools for experiments using recombinant proteins.

A confirmation of antibody reactivity with the endogenous protein is essential. There are many different ways to achieve this. A comparison with the published distribution of the protein in the literature is helpful, but this is not available for all proteins and sometimes the existing data may be inaccurate. It may, for example, have been generated using a poor-quality antibody. Thus, combining approaches to test antibody binding to the endogenous protein with those to determine the specificity of this recognition is advisable, and a combined approach is described below.

### STEP 4B. Antibody specificity for the target antigen

Reactivity with the endogenous protein does not in itself guarantee that recognition of the target antigen is specific. There are, furthermore, also degrees of specificity; some antibodies may only specifically detect their target antigen in particular tissues (e.g., those that lack expression of a cross-reactive protein) or in particular technical applications. While absorption tests using the immunising peptide to block antibody binding can be useful technical controls to detect non-specific staining (only of the secondary antibody in the case of monoclonal antibodies because all binding by the single epitope will be blocked by the immunogen), such tests cannot prove that an antibody is specific for target antigens that share common epitopes. We thus do not recommend this step for validating an antibody.

Some degree of likely cross-reactivity with related proteins can be predicted based on the sequence homology of the target antigens with other molecules. While the ideal situation is to avoid antibodies against these regions, this is not always possible. When validating an antibody where the immunogen contains significant homology to other related proteins, then it will be necessary to test the specificity of the reagent against the other proteins. An example of this was the BCL11A_XL_ antibody (clone BCL11A/123, Banham/Pulford University of Oxford), which was tested for cross reactivity against BCL11B as these molecules shared significant homology within the C-terminal immunogen.^27^ Antibodies can also exhibit cross-reactivity to epitopes (e.g., those that share a common conformation, epitope mimicry) that are not predictable based on sequence analysis. An ideal situation would be testing of several different antibodies in parallel because common patterns of antibody reactivity can substantially strengthen confidence in the validation data that are generated. Additional validation should be undertaken when using only a single antibody that shows unexpected patterns of reactivity compared to expression of its transcript or other sources of information.

Ideally, start by testing antibody reactivity (usually in multiple techniques and preferably with more than one antibody) with at least 2 positive and 2 negative cell lines or tissues (usually transcript +/−), including the cell type proposed for study. Antibodies will need to be titrated to obtain the optimal dilution to enable sensitive detection while minimizing non-specific background binding (compared to an isotype-matched negative control antibody). Working dilutions commonly vary both between different laboratories and across different techniques; a useful guide is available at www.abcam.com/protocols/antibody-dilutions-and-titer. Consider that post-transcriptional regulation of protein expression, e.g., posttranslational modifications or assembly into multi-protein complexes, may occasionally cause failure to detect protein where transcripts are detectable. If antibodies work in WB, then this technique is usually routinely included for antibody validation because it provides useful data on the molecular weight of the target antigen and whether multiple isoforms exist.

Silencing the expression of the target antigen *ex vivo* in cell lines by siRNA or shRNA, (and more recently using CRISPR/Cas9 technology), is now more widely being employed as a method for confirming the specificity of endogenous antigen recognition, particularly for validating antibodies against human proteins where *in vivo* manipulation cannot be performed. However, care must be taken, as silencing a single exon may not be sufficient to eliminate all transcripts that encode the target antigen. If the antibody recognizes the murine protein, then knock out (KO) mice provide a useful system to test reactivity with the endogenous protein, as long as this is accompanied by loss of the target antigen/epitope and not just loss of function, e.g., by truncation. However, difficulties can also arise when using silencing or KO approaches if the target antigen also regulates other closely related proteins. For example, the circadian regulator Bmal1 was found to regulate expression of the related *Bmal2* gene,^28^ resulting in a functionally double Bmal1 and Bmal2 KO, which could not have been used to address antibody specificity for distinguishing reactivity against Bmal1 vs. Bmal2. Another pitfall arose when characterizing an antibody where the immunogen was mammalian cells transfected with a transcription factor. Initial antibody validation looked promising, until it became apparent that, although the reagent recognized only transfected cells by immunohistochemistry, the subcellular distribution of the epitope tagged recombinant target antigen was different to that of the endogenous protein recognized by the monoclonal antibody. Further validation suggested that the antibody recognized a protein whose expression was induced by its transcriptional regulator (unpublished data).

Antibody validation files available on the EuroMAbNet website (www.euromabnet.com/monoclonal-antibodies) provide further examples of how antibodies have been validated through different technical applications and across species.

### STEP 5: Reproducibility and dissemination of data

In practice, it is important that antibodies are independently evaluated by multiple users and that the results are disseminated throughout the research community. While this activity does occur, it is generally on a fairly small scale, such as individual research groups reporting experiences with a particular reagent, comparing the reactivity of a panel of antibodies to a particular molecule or user feedback to a particular company or personal communication to the academic supplier. There are increasingly suggestions that there should be more standardization and several platforms have been proposed for sharing antibody validation data.

AbMiner was developed as a relational web-based database freely providing information on more than 600 commercial antibodies that were validated by WB for protein microarray studies.^29^ While this offers information, it does not seem to engage end users in reporting their own data. The same comment applies to the wealth of data available in the Human Protein Atlas (much of which is generated using only partially validated reagents), which does not link antibodies with externally published validation or provide a mechanism for user feedback. The Human Protein Atlas team have released data completing the first draft of the human proteome (www.proteinatlas.org/humanproteome).

Antibodypedia was developed within the 6^th^ framework EU program ProteomeBinders and the Human Proteome Organization's Human Antibody Initiative. This site has been launched as a more general portal for sharing antibody and antigen validation data.^30^ Antibodies available to the public from commercial or academic providers can be submitted, along with application-specific data that rates them against standard validation criteria as supportive, uncertain or non-supportive. Users can submit their own validation data and can also submit comments about the use of a particular antibody, to ensure sharing of both positive and negative findings. While this site does contain a large number of entries, it appears that these are primarily from a fairly limited number of commercial antibody suppliers and the Protein Atlas. The aim of the European ProteomeBinders consortium is to focus on quality-controlled replenishable protein affinity reagents (PARs) for the whole human proteome.^31^ This consortium has already proposed a Proteomics Standards Initiative (PSI)-PAR as a global community standard format for the representation and exchange of protein affinity reagent data^32^ that will be adopted by Antibodypedia. Recently another Antibody Validation Database website was established by a consortium testing >200 antibodies raised against 57 different histone modifications: more than 25% of the antibodies failed specificity tests by dot blot or WB, and, of the remainder, more than 20% failed in ChIP experiments.^21^ Although this site initially only specifically addressed histone-modification antibodies tested from the ENCODE (Encyclopedia of DNA Elements) and Roadmap Epigenomics projects, there are plans to add new non-histone antibodies from these projects. Importantly the site also invites other researchers to upload their validation data. ProteinSimple is also user-interactive and reports data from antibody screening using simple Western charge- and size-based assays.

## Summary and Conclusions

It seems to be fairly universally accepted that there is a need for standardization of antibody validation and for easy public access to validation data to help researchers independently choose the most specific and effective reagents. How exactly these goals will be achieved will probably be determined primarily by the large consortiums generating reagents to characterize the human proteome. We hope that our contribution will help many researchers through the maze of antibody validation and enable them to appreciate the experimental nature of these reagents, and to approach their use with a degree of healthy scepticism. Research to explore the human proteome and deliver personalised medicine will continue to fuel the demand for greater numbers of antibodies with increasing specificity and functional activity.
